# Dynamic changes in DNA modification states during late gestation male germ line development in the rat

**DOI:** 10.1186/1756-8935-7-19

**Published:** 2014-08-07

**Authors:** Catherine M Rose, Sander van den Driesche, Richard M Sharpe, Richard R Meehan, Amanda J Drake

**Affiliations:** 1 Endocrinology Unit, University/BHF Centre for Cardiovascular Science, University of Edinburgh, The Queen's Medical Research Institute, 47 Little France Crescent, Edinburgh EH16 4TJ, UK; 2 MRC Centre for Reproductive Health, University of Edinburgh, The Queen's Medical Research Institute, 47 Little France Crescent, Edinburgh EH16 4TJ, UK; 3 MRC Human Genetics Unit, IGMM, Western General Hospital, Crewe Road, Edinburgh EH4 2XU, UK

**Keywords:** Germ cells, Rat, DNA modification, 5-methylcytosine, 5-hydroxymethylcytosine, 5-formylcytosine, 5-carboxylcytosine, Thymine DNA Glycosylase

## Abstract

**Background:**

Epigenetic reprogramming of fetal germ cells involves the genome-wide erasure and subsequent re-establishment of DNA methylation. Mouse studies indicate that DNA demethylation may be initiated at embryonic day (e) 8 and completed between e11.5 and e12.5. In the male germline, DNA remethylation begins around e15 and continues for the remainder of gestation whilst this process occurs postnatally in female germ cells. Although 5-methylcytosine (5mC) dynamics have been extensively characterised, a role for the more recently described DNA modifications (5-hydroxymethylcytosine (5hmC), 5-formylcytosine (5fC) and 5-carboxylcytosine (5caC)) remains unclear. Moreover, the extent to which the developmental dynamics of 5mC reprogramming is conserved across species remains largely undetermined. Here, we sought to describe this process during late gestation in the male rat.

**Results:**

Using immunofluorescence, we demonstrate that 5mC is re-established between e18.5 and e21.5 in the rat, subsequent to loss of 5hmC, 5fC and 5caC, which are present in germ cells between e14.5 and e16.5. All of the evaluated DNA methyl forms were expressed in testicular somatic cells throughout late gestation. 5fC and 5caC can potentially be excised through Thymine DNA Glycosylase (TDG) and repaired by the base excision repair (BER) pathway, implicating 5mC oxidation in active DNA demethylation. In support of this potential mechanism, we show that TDG expression is coincident with the presence of 5hmC, 5fC and 5caC in male germ cell development.

**Conclusion:**

The developmental dependent changes in germ cell DNA methylation patterns suggest that they are linked with key stages of male rat germline progression.

## Background

**Figure 1:**
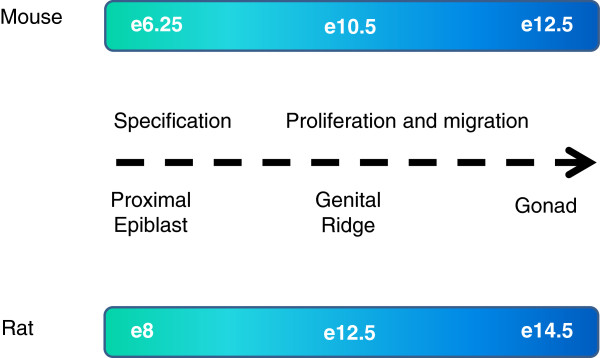
**Schematic diagram indicating timing of germ cell specification and migration in mouse and likely timings in the rat.** The timing of germ cell specification is well-described in the mouse in which PGCs specified from epiblast cells at around e6.25–7 migrate along the developing hind-gut endoderm (from e8.5) and colonise the genital ridges from e10.5. The process is less well-reported in the rat and probably occurs later than in the mouse [9].

Methylation of the cytosine base in DNA (DNA methylation) is an essential epigenetic mark in mammals that contributes to the regulation of transcription, chromatin organisation and histone modification deposition [1,2]. DNA methylation at regulatory regions, including promoters, is associated with stable transcriptional silencing of genes and transposons, genomic imprinting and X inactivation [3,4]. In order to give rise to functional gametes, primordial germ cells (PGC) undergo extensive epigenetic reprogramming including erasure of DNA methylation and extensive chromatin remodelling, a process which is thought to be necessary to remove potential epimutations and to erase parental imprints in this cell lineage [5-8]. In the mouse, PGCs specified from epiblast cells at around embryonic day (e) 6.25–7 migrate along the developing hind-gut endoderm (from e8.5) and colonise the genital ridges from e10.5 [8] (Figure 1). The precise timings in the rat are less well-documented but may occur slightly later than in the mouse (Figure 1) [9]. During mouse embryogenesis, DNA methylation is established by e6 and is thought to contribute to stable lineage commitment [10-12]. The erasure of DNA methylation in PGCs appears to be initiated from approximately e8.5 and is completed by approximately e13.5 [6,13,14] and occurs at the majority of the genome, with the exception of certain loci, some of which correspond to retrotransposons [15,16]. Erasure of DNA methylation is followed by a period of remethylation, the timing of which differs between the male and female germlines. In the mouse, DNA remethylation is initiated at e15.5 and continues until e18.5 and beyond (at least at imprinted genes and repeats) in male germ cells, whereas in females, the process occurs after birth [14,16-21]. Although epigenetic reprogramming has been extensively investigated in mouse germ cells, the extent to which this process is conserved across species remains largely undetermined. Notably, the degree of active demethylation of cytosine methylation in the sperm genome prior to forming a functional zygotic nucleus varies in different mammals; for example, demethylation is extensive in the mouse, but in contrast, there is no observable demethylation of the sheep male pronucleus at any point in the first cell cycle [22-24].

The potential pathways governing active removal of 5-methylcytosine (5mC) are now being elucidated with the recent discovery of further modified forms of cytosine nucleotides including 5-hydroxymethylcytosine (5hmC), 5-formylcytosine (5fC) and 5-carboxylcytosine (5caC), which are sequentially produced from 5mC by the action of the ten-eleven translocase (TET) family of iron, abscorbic acid and α-ketoglutarate-dependent dioxygenases [25-29]. 5hmC-modified CpGs are significantly enriched at the bodies of actively transcribing genes in many tissues (but not all) and are present to some degree at enhancer elements and a small cohort of regions spanning an annotated transcriptional start site [30-34] whilst the levels of 5fC and 5caC are low in somatic cells, perhaps in part due to their potential inhibitory effect on transcription [35]. Although the dynamics of 5mC, 5hmC, 5fC and 5caC have been investigated in fertilised zygotes from a variety of organisms [36-40], there are few reported studies for developing fetal germ cells [13]. A recent study has shown that DNA demethylation in mouse PGCs entails conversion of 5mC to 5hmC by TET1 and TET2 with the loss of 5mC largely complete by e11.5 [13]. The progressive decline in 5hmC in the absence of enrichment of 5fC and 5caC suggests that subsequent demethylation may occur by a replication-dependent mechanism in the mouse. Any role for 5hmC, 5fC and/or 5caC in the later stages of germline epigenetic reprogramming is not known.

Here, we have investigated the spatiotemporal relationship between 5mC, 5hmC, 5fC and 5caC specifically during late (e14.5–e21.5) male fetal rat germ cell development by immunofluorescence using previously validated antibodies [13,14,41-44]. Although the timing of germ cell specification is not as well described in the rat as in the mouse, germ cells are present in the rat embryonic testis by e14.5 (Figure 1). We firstly wished to address if male germ cell development in the rat is associated with global demethylation and subsequent remethylation as in the mouse and, secondly, if 5hmC, 5fC and 5caC are present in a pattern that is indicative of a developmental-associated function in the rat germline. Since 5fC and 5caC can both be ‘repaired’ by Thymine DNA Glycosylase (TDG) to produce unmodified cytosine, we also sought to assess whether TDG was present in the rat germline during late fetal development to potentially mediate active DNA demethylation [26,45].

## Results

### Immunohistochemistry for 5mC and DAZL

**Figure 2:**
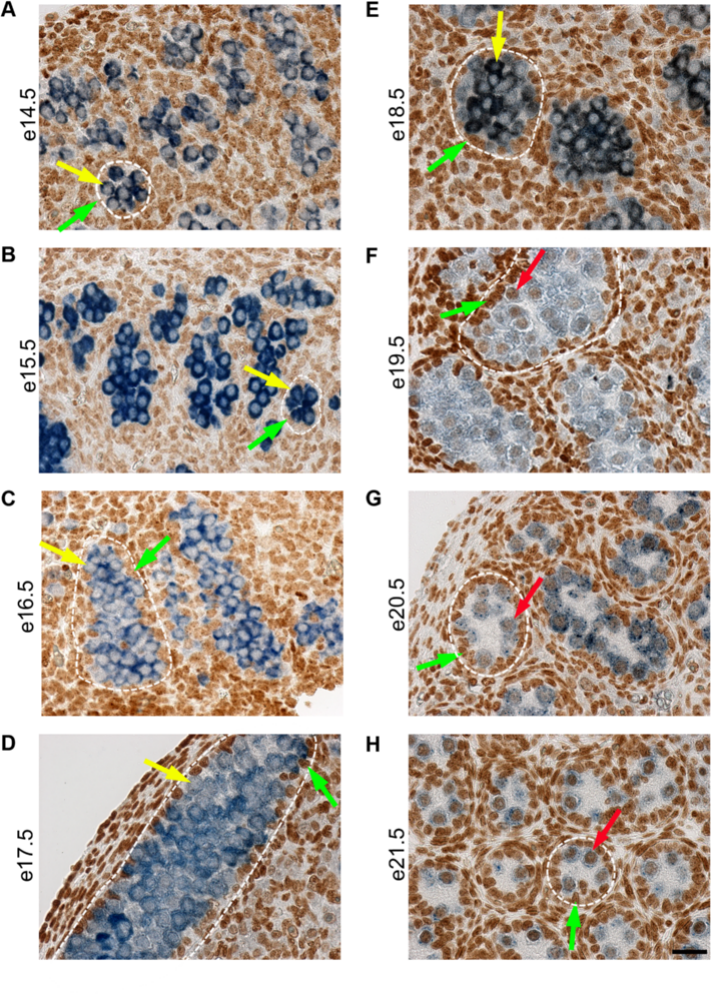
**Immunohistochemistry showing localisation of germ cells and 5mC during mid to late gestation.** In order to clearly show the localisation of germ cells at all stages of development, we used the specific germ cell cytoplasmic marker DAZL, which survives acid denaturation, in combination with 5mC for immunohistochemistry. The cytoplasm of germ cells is stained for DAZL in blue and 5mC is indicated by brown staining in nuclei. Germ cells (indicated by yellow arrows) are located within seminiferous cords which are surrounded by somatic cells; one seminiferous cord is shown outlined in each image. There are also somatic cells within the seminiferous cords (green arrows). Images show that 5mC is undetectable in germ cells between e14.5 and e18.5 **(A** to **E)**. 5mC is detectable in germ cells at e19.5 (**F**, indicated by red arrow) and is more marked in germ cells at e20.5 and e21.5 **(G, H)**. 5mC is present in somatic cells throughout the time course. Scale bar = 50 μm.

Several commonly used germ cell markers are not compatible with the hydrochloric acid (HCl) antigen retrieval of modified cytosines for use in immunofluorescence (IF). In order to clearly show the localisation of 5mC to germ cells at all stages of development, we used the specific germ cell cytoplasmic marker DAZL, which survives HCl antigen retrieval, in combination with 5mC for immunohistochemistry (IHC) (Figure 2). The testis contains different cellular compartments; germ cells (shown with cytoplasm stained for DAZL in blue) are rounded in shape and contained within the seminiferous cords, which also contain Sertoli cells (Figure 2). The hormone-producing Leydig cells, peritubular myoid cells and other interstitial cell types are located outside of the seminiferous cords in the interstitium. There was no/little detectable 5mC in germ cells between e14.5 and e18.5 (Figure 2A,B,C,D,E), suggesting that, as in mice, rat germ cells are hypomethylated relative to surrounding somatic cells by e14.5. 5mC was detectable in some germ cells from e19.5 onwards, and by e21.5, there was strong 5mC immunostaining (Figure 2F,G,H). Again, this is in line with mouse studies in which remethylation occurs in male germ cells during late gestation [5].

### Immunofluorescence for 5mC, 5hmC, 5fC, 5caC and TDG

**Figure 3:**
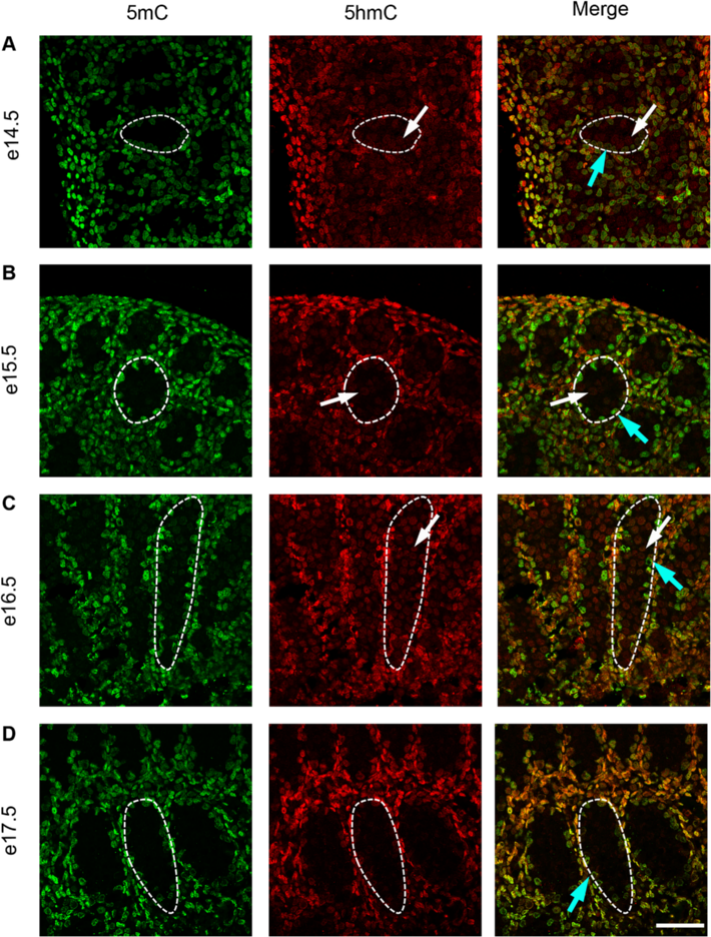
**Immunofluorescence showing localisation of 5mC (green) and 5hmC (red) between e14.5 and e17.5.** One seminiferous cord is shown outlined in each image; germ cells are indicated by white arrows and somatic cells within the seminiferous cord are indicated by blue arrows. Images show that 5mC (green) is undetectable in germ cells between e14.5 and 17.5 **(A** to **D)**. In contrast, 5hmC is detectable in germ cells between e14.5–e16.5 **(A** to **C)** (red, arrows) but is absent by e17.5 **(D)**. Both forms of methylation are found in somatic cells throughout the time course. Scale bar = 50 μm.

**Figure 4:**
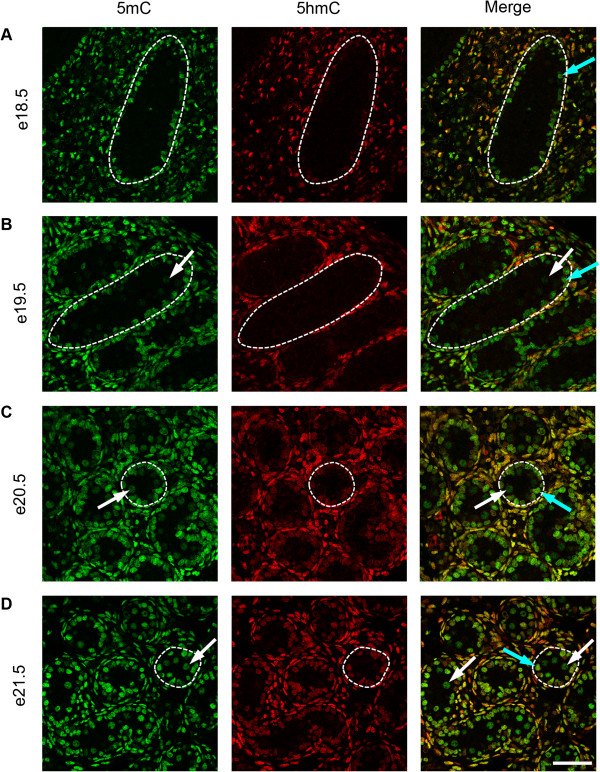
**Immunofluorescence showing localisation of 5mC (green) and 5hmC (red) between e18.5 and e21.5.** One seminiferous cord is shown outlined in each image; germ cells are indicated by white arrows, and somatic cells within the seminiferous cord are indicated by blue arrows. Images show that 5mC (green) is undetectable in germ cells at e18.5 **(A)** but becomes detectable at e19.5 **(B)** (indicated by arrows) and is more marked in germ cells at e20.5 and e21.5 **(C, D)**. In contrast, 5hmC is undetectable in germ cells between e18.5–e21.5 (red, arrows; **A** to **D)**. Both forms of methylation are found in somatic cells throughout the time course. Scale bar = 50 μm.

**Figure 5:**
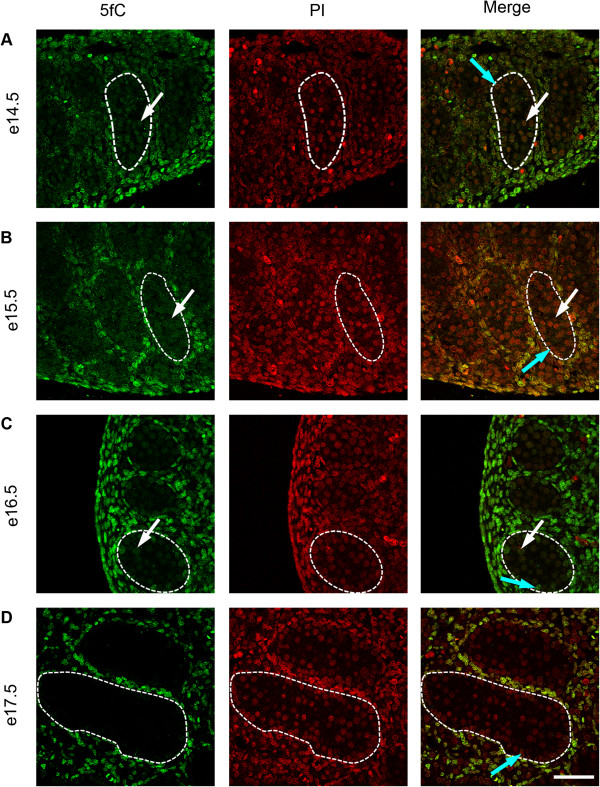
**Immunofluorescence showing localisation of 5fC between e14.5 and e17.5.** One seminiferous cord is shown outlined in each image; germ cells are indicated by white arrows and somatic cells within the seminiferous cord are indicated by blue arrows. Images show that 5fC is present in germ cells between e14.5–e16.5 **(A** to **C)** but is not detectable at e17.5 **(D)**. Propidium iodide was used as a nuclear counterstain (red). 5fC is detectable in somatic cells throughout the time course. Scale bar = 50 μm.

**Figure 6:**
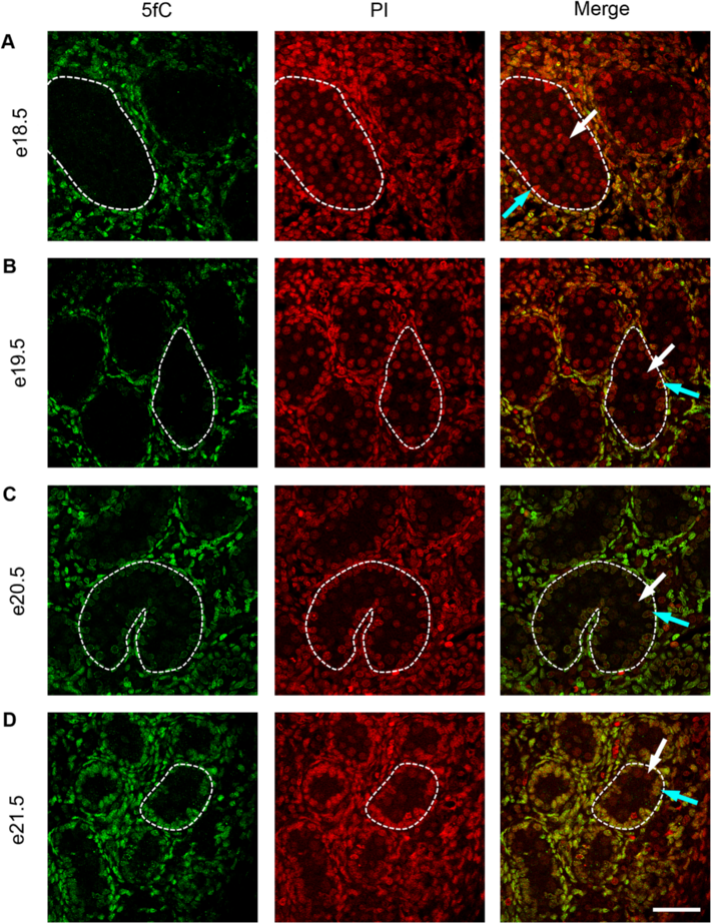
**Immunofluorescence showing localisation of 5fC between e18.5 and e21.5.** One seminiferous cord is shown outlined in each image; germ cells are indicated by white arrows and somatic cells within the seminiferous cord are indicated by blue arrows. Images show that 5fC is undetectable in germ cells between e18.5–e21.5 **(A** to **D)**. Propidium iodide was used as a nuclear counterstain (red). 5fC was detectable in somatic cells throughout the time course. Scale bar = 50 μm.

**Figure 7:**
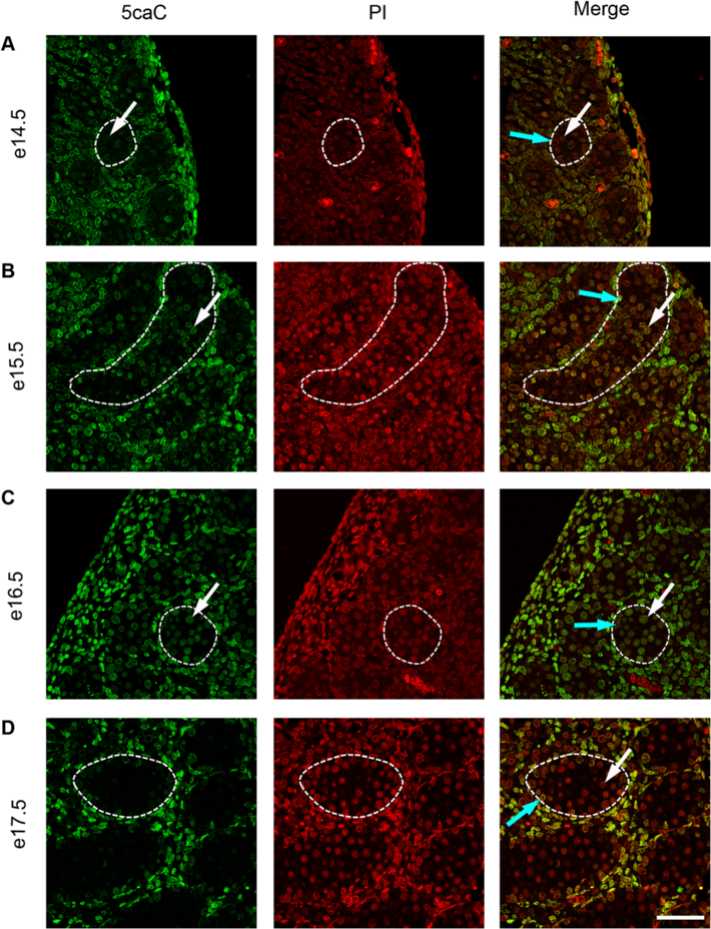
**Immunofluorescence showing localisation of 5caC between e14.5 and e17.5.** One seminiferous cord is shown outlined in each image; germ cells are indicated by white arrows and somatic cells within the seminiferous cord are indicated by blue arrows. Images show 5caC is present in germ cells between e14.5–e16.5 **(A** to **C)** but is not detectable at e17.5 **(D)**. Propidium iodide was used as a nuclear counterstain (red). 5caC was detectable in somatic cells throughout the time course. Scale bar = 50 μm.

**Figure 8:**
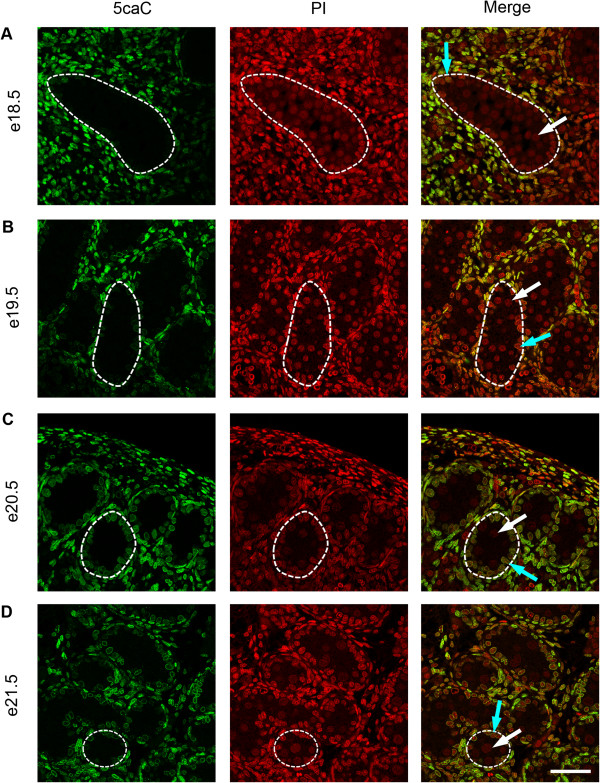
**Immunofluorescence showing localisation of 5caC between e18.5 and e21.5.** One seminiferous cord is shown outlined in each image; germ cells are indicated by white arrows and somatic cells within the seminiferous cord are indicated by blue arrows. Images show that 5caC is undetectable in germ cells between e18.5-e21.5 **(A** to **D)**. Propidium iodide was used as a nuclear counterstain (red). 5caC was detectable in somatic cells throughout the time course. Scale bar = 50 μm.

**Figure 9:**
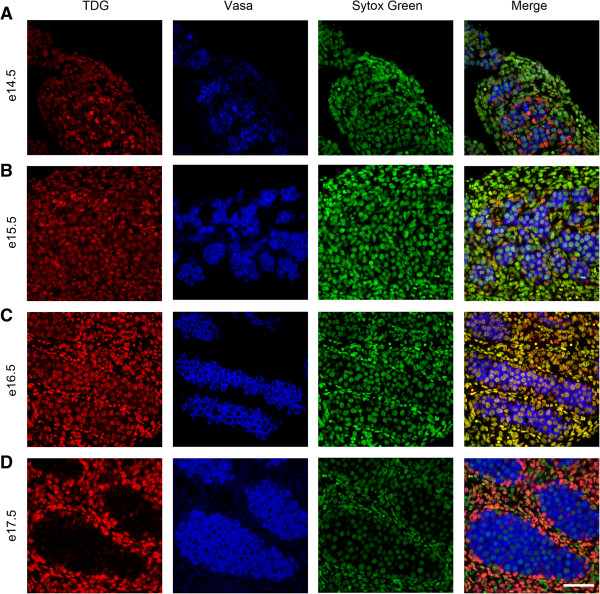
**Immunofluorescence showing localisation of TDG between e14.5 and e17.5.** TDG is stained in red, the cytoplasm of germ cells is stained for VASA in blue and nuclei are stained using sytox green (green). Images show TDG is present in germ cells between e14.5–e16.5 **(A** to **C)** but is not detectable at e17.5 **(D)**. TDG is detectable in somatic cells throughout the time course. Scale bar = 50 μm.

**Figure 10:**
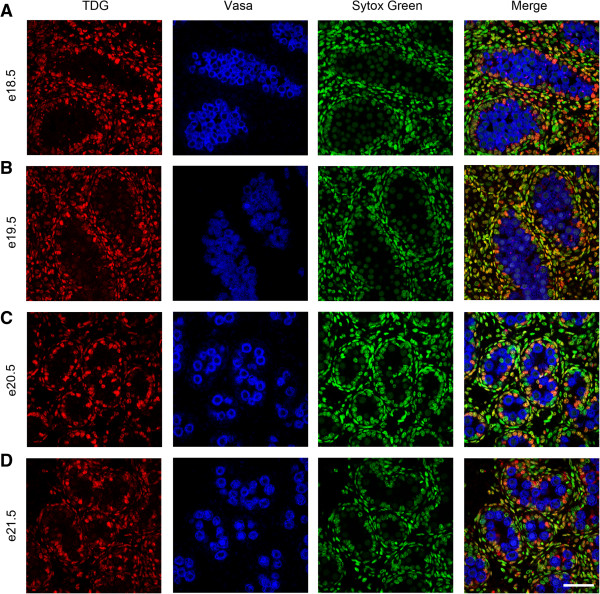
**Immunofluorescence showing localisation of TDG between e18.5 and e21.5.** TDG is stained in red, the cytoplasm of germ cells is stained for VASA in blue and nuclei are stained using sytox green (green). Images show TDG is largely undetectable in germ cells between e18.5–e21.5 **(A** to **D)**. TDG is detectable in somatic cells throughout the time course. Scale bar = 50 μm.

To investigate the temporal expression patterns for germ cell 5mC, 5hmC, 5fC and 5caC, immunofluorescence was used. Consistent with the IHC findings, little 5mC was detectable in germ cells between e14.5 and e18.5 (Figure 3A,B,C,D and Figure 4A), but 5mC was detectable in some germ cells at e19.5, with enhanced staining at e21.5 (Figure 4B,C,D). In contrast, 5hmC showed some germ cell localisation between e14.5–e16.5 (Figure 3A,B,C) but had become undetectable by e17.5 and remained undetectable up to e21.5 (Figure 3D and Figure 4A,B,C,D). Semi-quantitative analysis of the images confirmed a significant decrease in 5mC and an increase in 5hmC between e16.5 and e21.5 (Additional file 1: Figure S1). Similarly, 5fC was detectable in germ cells at e14.5–e16.5 (Figure 5A,B,C) but not after this age (Figure 5D and Figure 6A,B,C,D). 5caC was also detectable in germ cells only at e14.5–e16.5 (Figure 7A,B,C) but not at later ages (Figure 7D and Figure 8A,B,C,D). In contrast to the germ cells, 5mC and 5hmC in somatic cells was omnipresent and appeared stable throughout late gestation. The findings are summarised in Table 1. The absence of detectable 5mC from e14.5 onwards until e19.5 and the limited appearance (between e14.5–e16.5) of 5hmC, 5fC and 5caC might suggest that the oxidised forms of 5mC are part of a demethylation process that is complete by e16.5. It is unclear if this is indicative of an active or a passive demethylation pathway during rat male germ cell development. We therefore investigated the pattern of TDG staining in developing fetal germ cells. The protocol for TDG staining was compatible with protocols for counterstaining for germ cell cytoplasm (VASA) and nucleus (sytox green) (Figure 9). TDG was clearly detectable in VASA positive germ cell nuclei between e14.5 and e16.5 (Figure 9A,B,C) but was largely undetectable at later ages (Figure 9D and Figure 10A,B,C,D). It is noteworthy that TDG expression in e14.5–e16.5 germ cells is coincident with the presence of 5hmC, 5fC and 5caC. In somatic cells, TDG was detectable at all stages examined.

**Table 1:** Detection of 5mC, 5hmC, 5fC, 5caC and TDG during mid to late gestation (e14.5–e21.5) in fetal rat germ cells

**Day**	**5mC**	**5hmC**	**5fC**	**5caC**	**TDG**
e14.5	−	+	+	+	+
e15.5	−	+	+	+	+
e16.5	−	+	+	+	+
e17.5	−	−	−	−	−
e18.5	−	−	−	−	−
e19.5	+ in a subset of germ cells	−	−	−	−
e20.5	+	−	−	−	−
e21.5	+	−	−	−	−

5mC, 5hmC, 5fC and 5caC are detectable in somatic cells throughout this time-course. + indicates presence, and − indicates absence of staining.

## Discussion

Our study represents a novel characterisation of the dynamics of DNA modification of fetal germ cells in the male rat during the latter half of gestation. The lack of detectable 5mC in germ cells at e14.5 suggests that the process of 5mC erasure is already complete by this time. This is consistent with data in mice showing that DNA methylation is erased during PGC migration [5,14,42] such that by e13.5, the overall level of DNA methylation is reduced by more than 90% [7].

One proposed mechanism for DNA demethylation in fetal germ cells is TET-mediated 5mC to 5hmC conversion [13]. *In vitro* PGC (iPGC) formation and genome-wide DNA demethylation are unaffected by the absence of 5hmC mediated by TET1 and TET2 [46]. However, this leads to hypermethylation in many target loci in mutant iPGCs, consistent with a role for 5hmC as an intermediate in locus-specific demethylation. In the mouse germline, the loss of 5mC in exons, at promoters and imprinted regions is associated with an increase in 5hmC which is mostly completed by e11.5, followed by a progressive decline over several cell cycles between e11.5 and e13.5, suggesting a replication-dependent mechanism for 5hmC loss [13]. Our study, showing the stable persistence of 5hmC in fetal rat germ cells until e16.5, suggests that the removal of 5mC through conversion to 5hmC might also occur in the rat. 5fC and 5caC were also present in fetal germ cells between e14.5 and e16.5, but, as with 5hmC, were no longer present at e17.5. Recent data has shown enrichment of 5hmC in mouse PGCs during the phase of DNA demethylation [13,42], with a progressive later decline; however, these studies were undertaken using Oct4-GFP transgenic mice which allow the isolation of migratory germ cells from urogenital ridges at an early stage and such studies are not yet possible in the Wistar rat. We did not observe enrichment of 5hmC in rat germ cells relative to the surrounding somatic cells between e14.5 and e16.5, and this may indicate that this occurs before e14.5 in the rat and that 5hmC levels have already begun to decline by this time, as in the mouse [13,42]. The lack of enrichment of germ cell 5fC and 5caC and the presence of these modifications in surrounding somatic cells has previously been reported, at least up until e12.5 in the mouse [13,42].

Given their proposed role in the TET-mediated demethylation process, the presence of 5fC and 5caC in fetal germ cells could reflect an accumulation of unconverted intermediates during global demethylation before rapid removal between e16.5 and e17.5. Although little is known regarding the function of 5fC and 5caC, a recent study showing that the levels of these modifications gradually decrease with cell division in the mouse zygote, rather than exhibiting the rapid removal that might be expected if they existed solely as part of a demethylation mechanism, indicates that they may also have a functional role in chromatin remodelling [37]. Thus, the stable presence of 5hmC, 5fC and 5caC in rat germ cells between e14.5 and e16.5, and in testicular somatic cells throughout late gestation, may indicate potential functionality of these modifications during germ cell development.

TET-mediated iterative oxidation of 5mC and 5hmC to 5fC and/or 5caC may provide suitable substrates for demethylation to non-modified cytosine via the rapid excision of 5fC and 5caC by TDG, followed by base excision repair (BER) [29,37,45,47]. Support for this comes from comparative analysis of 5hmC/5fC/5caC distributions in wild-type and TDG-deficient mouse ES cells showing that a large number of genomic loci are targeted by TET/TDG activities [48] and from recent work suggesting that 5fC is detectably enriched over the bodies of actively transcribing genes as well as over CpG islands and promoters in ES cells, and that these levels increase in the absence of TDG activity [43]. Consistent with a role for TDG in germ cell DNA demethylation in the rat, TDG was present in germ cells between e14.5 and e16.5, but largely absent thereafter, when 5fC and 5caC were no longer detectable. There are, however, alternative mechanisms for DNA demethylation including through deamination of 5mC to thymine by an activation-induced deaminase/apolipoprotein B editing complex (AID/APOBEC), resulting in a G/T mismatch that is subsequently converted to G/C by TDG and subsequent BER. Support for this comes from data showing that AID-deficient PGCs have substantially delayed demethylation (although loss of 5mC still occurs) [49,50]. Recent studies in the mouse zygote show that there are two phases of active DNA demethylation with involvement of TET3 and, independently, AID-mediated cytosine deamination and subsequent BER in the second phase whilst the mechanisms accounting for DNA demethylation in the first phase remain unclear [41]. Further clarification of the roles of AID- and/or TDG-mediated demethylation in PGCs is still required.

The re-establishment of 5mC in male rat germ cells appears to begin at e19.5. The remethylation phase has been less well characterised in mouse germ cells, although gene-targeted studies suggest that in male germ cells, the onset of DNA remethylation occurs at some imprinted loci and at repetitive elements from e15.5, with global remethylation re-established by e18.5 [51,52] which may be further augmented perinatally [17,18]. Our demonstration of the absence of 5hmC, 5fC and 5caC in rat germ cells during the re-establishment of DNA methylation between e19.5 and e21.5 further supports the hypothesis that these variants form part of the initial demethylation pathway in the male germline [29].

## Conclusions

This study provides the first characterisation of global patterns of DNA methylation in the fetal rat testis for all currently recognised forms of cytosine methylation during mid to late gestation and provides an insight into the dynamics of remethylation in male rat fetal germ cells. The timing of both erasure and re-establishment of DNA methylation is later than that in the mouse, consistent with the longer gestation period in the rat (22 days in Wistar rats, compared to approximately 19 days in the mouse [53]). The extent to which germline reprogramming operates in other species is currently poorly understood. However, studies for example in pigs indicate that epigenetic reprogramming in germ cells follows the same dynamics as in mice, although the phase of demethylation occurs over a period of 20 days [54]. There are few published studies in humans, but data suggests that in human males, germ cell DNA is hypomethylated in mid-gestation and 5mC levels increase from around 20 weeks to term [55,56]. Levels of 5hmC, 5fC and 5caC decrease as human spermatogenesis proceeds, whilst 5mC levels remain constant [57]. As in mouse, this suggests that during spermatogenesis active DNA demethylation mechanisms are down-regulated, stabilising methylation profiles in mature sperm, which can be subsequently reprogrammed in fertilised zygotes [58]. Our study, demonstrating the dynamics of epigenetic reprogramming in fetal germ cells in the rat, provides further evidence that this is conserved across species. The stable presence of 5hmC, 5fC and 5caC in germ cells for several days following the loss of 5mC also support data from the pig suggesting that epigenetic reprogramming may occur over an extended period in species with longer gestation [54]. Although altered epigenetic reprogramming early in gestation during the period of DNA methylation erasure has been proposed as one mechanism accounting for transgenerational inheritance in mammals [14], we and others have proposed that disruption of epigenetic reprogramming in the second half of gestation could also be important in the transmission of environmentally induced effects across generations through the male line [59-64]. Our study therefore also provides a baseline for investigating the susceptibility of this process to disruption, for example as a consequence of exposure to environmental factors.

## Methods

### Animals

#### Ethics statement

These studies were specifically approved by the UK Home Office and were conducted under an approved Project Licence (PPL 60/3914) in accordance with the UK Animals (Scientific Procedures) Act 1986 following review by the University of Edinburgh Animal Research Ethics Committee. Wistar rats were maintained in our own facility in an environment of controlled humidity, temperature (22°C), lighting (artificial light between 7.00 a.m.–7.00 p.m.), and constant access to breeding diet (RM3(E) soya free; Special Diets Services, Witham, Essex, UK) and water. Females were time mated and killed by CO_2_ asphyxiation and subsequent cervical dislocation at experimental time points between e14.5 and e21.5 (gestation is approximately 22 days in our colony). Fetuses were removed, decapitated, and placed in ice cold phosphate-buffered saline (PBS, Sigma-Aldrich, Dorset, England, UK). Testes were microdissected and incubated in Bouin's fixative for 1 h at room temperature. Tissues were then embedded in paraffin wax following standard procedures and 5 μm sections prepared.

### 5mC and DAZL immunohistochemistry

Tissue sections were de-waxed and rehydrated before submersion in Novocastra Epitope Retrieval Solution (pH9, Leica, Milton Keynes, UK) and pressure cooking for 5 min at 125°C. Endogenous peroxidase activity was then blocked by incubation in 3% (*v*/*v*) hydrogen peroxide/methanol. All subsequent washes were in TBS, and antibody/serum incubations were conducted in a humidity chamber (Fisher Scientific, Loughborough, Leicestershire, UK). Tissues were blocked with normal goat serum (NGS, Biosera, East Sussex, UK) diluted 1:5 with 5% (*w*/*v*) BSA in TBS (NGS/TBS/BSA) before incubation with DAZL antibody (1:500, mouse, AbD Serotec, Oxford, UK) overnight at 4°C. Slides were then washed and incubated with goat anti-mouse biotinylated antibody (1:500, Vector Laboratories, Burlingame, CA, USA) for 30 min. Slides were washed before incubation with Streptavidin-Alkaline Phosphatase (1:200, Vector Laboratories) for 30 min. Following further washing, antibodies were detected using PermaBlue Plus/AP (Diagnostic Biosystems, Pleasanton, CA, USA) following manufacturer's instructions. Tissues were incubated for 15 min in 4 M hydrochloric acid (HCl)/TBS, preheated to 37°C, then washed with 0.1% Tween in TBS. Tissue was permeabilised with 1% Triton X-100 (Sigma-Aldrich, St. Louis, MO, USA) in TBS for 30 min before blocking with normal horse serum (NHS, Biosera) diluted 1:5 with 5% (*w*/*v*) BSA in TBS (NHS/TBS/BSA). Slides were incubated with 5mC antibody (1:300, mouse, Eurogentec, London, UK) overnight at 4°C. Slides were then washed and antibody detected using the ImmPress anti-mouse Ig (peroxidase) Polymer Detection Kit (Vector Laboratories) and ImmPress Diaminobenzidine (DAB, Vector Laboratories) following manufacturers' instructions. Mounting was conducted with PermaFluor Aqueous Mounting Medium (Thermo Fisher Scientific, Waltham, MA, USA).

### Immunofluorescence for 5mC and 5hmC

For immunofluorescence, following initial preparation as above, tissues were blocked with NGS (Biosera) diluted 1:5 with 5% (*w*/*v*) BSA in TBS (NGS/TBS/BSA) before incubation with 5mC antibody (1:100, mouse, Eurogentec) overnight at 4°C. Slides were washed before the addition of goat anti-mouse biotinylated secondary antibody (1:500, Dako, Berkshire, UK), subsequently detected during incubation for 60 min with Alexa Fluor 488 streptavidin (1:200, Invitrogen, Paisley, UK). For detection of 5hmC, following further washing, tissues were again blocked with NGS/TBS/BSA and incubated with 5hmC antibody (1:50, rabbit, Active Motif, Rixensart, Belgium) overnight at 4°C. Goat anti-rabbit Alexa Fluor 555 (1:200 in TBS, Invitrogen) was used to detect the primary antibody. All slides were mounted in PermaFluor Aqueous Mounting Medium (Thermo Fisher Scientific). Analysis was performed on three to ten fetal testes from three to six different litters for each modification at each time point and representative images captured as specified below. Semi-quantification of immunofluorescence was obtained using Image J Software (National Institute of Health). Each complete tubule in the image was analysed, identifying the region of interest as being within the seminiferous tubule, inside the ring of Sertoli cell nuclei. Intensity was expressed as mean pixel intensity for this region, normalised to the mean pixel intensity for somatic cells within the same image.

### Immunofluorescence for 5fC and 5caC

Following tissue preparation as above, tissues were blocked with NGS/TBS/BSA and incubated with either 5fC or 5caC antibodies (1:200 or 1:1,500, respectively, both Active Motif) overnight at 4°C. Antibodies were detected using goat anti-rabbit Alexa Fluor 555 (1:200) in TBS. For nuclear counterstaining, tissue was exposed to propidium iodide (Sigma-Aldrich) (1:500 in TBS) for 45 min before mounting. Analysis was performed on four to ten fetal testes from four to six different litters for each modification at each time point and representative images captured as specified below.

### Immunofluorescence for TDG

Following re-hydration, tissues were submerged in 0.01 M citric acid (sigma, pH6) and pressure cooked for 5 min at 125°C. Endogenous peroxidase activity was then blocked by incubation in 3% (*v*/*v*) hydrogen peroxide/methanol for 30 min. Tissues were blocked with normal chicken serum (NChS, Biosera) diluted 1:5 with 5% (*w*/*v*) BSA in TBS (NChS/TBS/BSA), before incubation with anti-TDG (1:500, rabbit, Sigma) antibody overnight at 4°C. Following washing, tissues were incubated with chicken anti-rabbit peroxidase-conjugated secondary antibody (1:200, Santa Cruz, CA, USA) in NChS/TBS/BSA for 30 min. Following further washing, slides were incubated with Tyramide-Cy3 (Perkin Elmer-TSA-Plus Cyanine 3 System, Perkin Elmer Life Sciences, Waltham, MA, USA) (1:50 in kit diluent) for 10 min. Following further washing, slides were microwaved at full power for 2.5 min in boiling 0.01 M citric acid before further blocking in NGS (Biosera) diluted 1:5 with 5% (*w*/*v*) BSA in TBS (NGS/TBS/BSA), then incubation with anti-Vasa (1:150, rabbit, Abcam, Cambridge, UK) antibody overnight at 4°C. Following further washing, tissue was incubated with goat anti-rabbit peroxidase-conjugated secondary antibody (1:200, Dako) in NGS/TBS/BSA for 30 min. Following further washing, slides were incubated with Tyramide-Cy5 (Perkin Elmer-TSA-Plus Cyanine 5 System, Perkin Elmer Life Sciences) (1:50 in kit diluent) for 10 min. Slides were washed and counterstained with sytox green (1:500 in TBS) for 30 min before mounting in Permafluor as previously.

### Image capture and processing

DAB immunohistochemistry was imaged using a Provis AX70 microscope (Olympus Optical, Southend-on-Sea, Essex, UK) and AxioCam HRc (Carl Zeiss Ltd., Cambridge, UK). An LSM 510 Meta confocal microscope (Carl Zeiss Ltd.) was used to image immunofluorescence, and all figures were produced using Photoshop CS5.1 (Adobe, San Jose, CA, USA).

## Abbreviations

5caC: 5-carboxylcytosine; 5fC: 5-formylcytosine; 5hmC: 5-hydroxymethylcytosine; 5mC: 5-methylcytosine; (e): embryonic day; AID/APOBEC: activation-induced deaminase/apolipoprotein B editing complex; BER: base excision repair; DAB: diaminobenzamine; IHC: immunohistochemistry; iPGC: *in vitro* primordial germ cells; PGC: primordial germ cells; TDG: Thymine DNA Glycosylase; TET: ten-eleven translocase.

## Competing interests

The authors declare that they have no competing interests.

## Authors' contributions

CR, RM and AD generated the main idea of the work and developed the study design. CR and SvdD acquired and analysed the data. AD, RM and RS contributed to analysis and interpretation of the data. RS and SvdD contributed materials. CM, RM and AD wrote the manuscript. SvdD and RS made comments, suggested appropriate modifications and made corrections. All authors read and approved the final manuscript.

## Supplementary Material

Additional file 1: Figure S1 Semi-quantification of immunofluorescence for 5mC and 5hmC at e16.5 and e21.5. Semi quantification of immunofluorescence was obtained using Image J Software and intensity expressed as mean pixel intensity for this region, normalised to the mean pixel intensity for somatic cells within the same image. There was a significant decrease in 5mC and an increase in 5hmC between e16.5 and e21.5. Click here for file
