# Supplementary figures and images for: Dynamic changes in DNA modification states during late gestation male germ line development in the rat
Source: Epigenetics Chromatin. 2014 Aug 7;7:19. doi: 10.1186/1756-8935-7-19 (PMC4163680; doi:10.1186/1756-8935-7-19)

## Slide 1
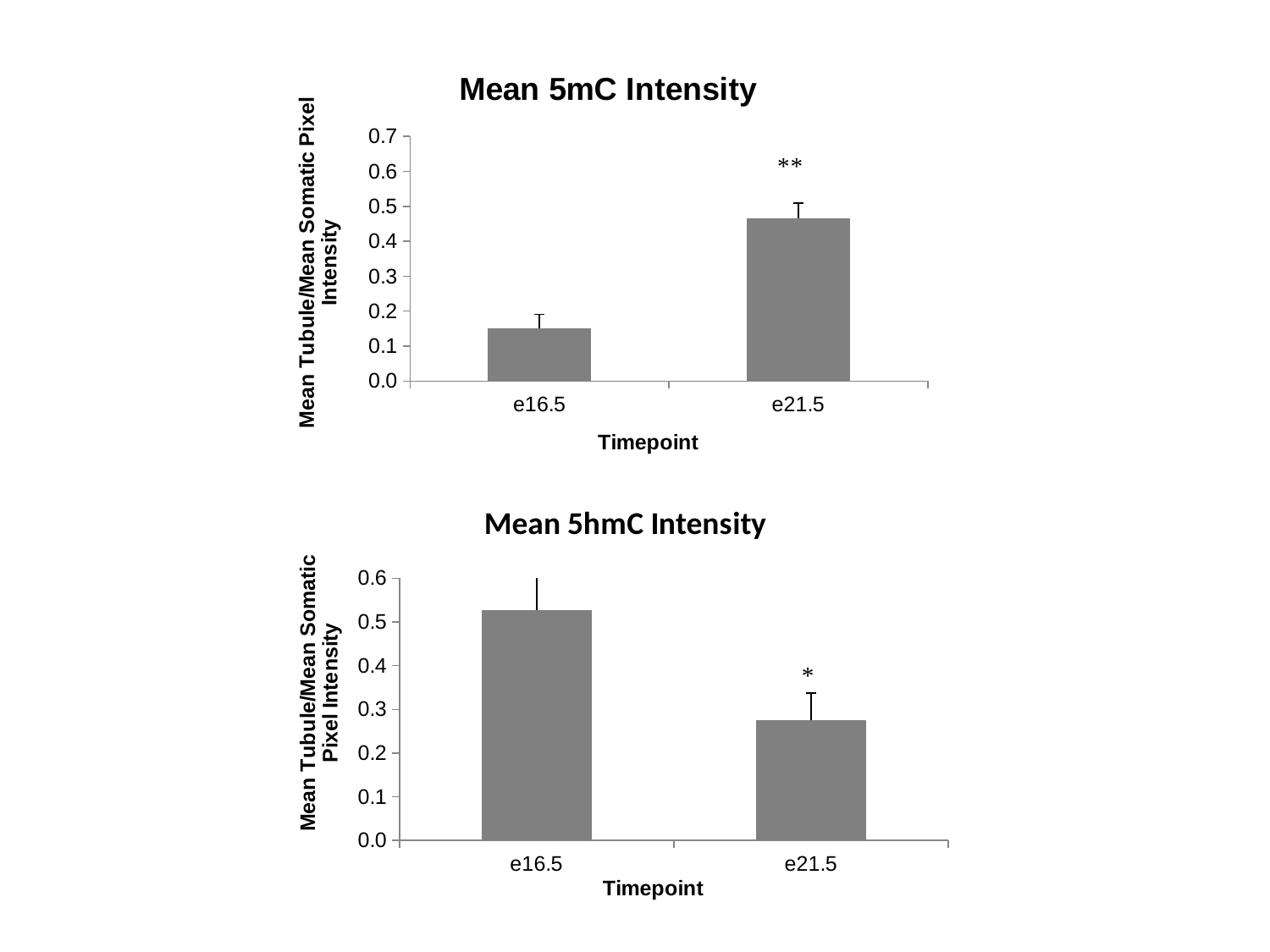

### Chart: Mean 5mC Intensity
| Category | |
|---|---|
| e16.5 | 0.15006233891039067 |
| e21.5 | 0.46446980475146665 |
### Chart: Mean 5hmC Intensity
| Category | |
|---|---|
| e16.5 | 0.5275274466749278 |
| e21.5 | 0.27550725446224533 |

Supplement: Additional file 1: Figure S1 — Semi-quantification of immunofluorescence for 5mC and 5hmC at e16.5 and e21.5. Semi quantification of immunofluorescence was obtained using Image J Software and intensity expressed as mean pixel intensity for this region, normalised to the mean pixel intensity for somatic cells within the same image. There was a significant decrease in 5mC and an increase in 5hmC between e16.5 and e21.5. [file 1756-8935-7-19-S1.pptx]
